# Advances in the study of oral microbiota and metabolism associated fatty liver disease: a systematic review

**DOI:** 10.3389/fcimb.2024.1491696

**Published:** 2024-11-12

**Authors:** Mingming Huang, Xinbi Zhang, Rui Zhou, Yingzhe Song, Jing Zhang, Jian Wu

**Affiliations:** ^1^ School of Kinesiology and Health, Capital University of Physical Education and Sports, Beijing, China; ^2^ Department of Hepatology, Beijing Youan Hospital, Capital Medical University, Beijing, China

**Keywords:** oral microbiota, oral microbiology, metabolism-associated fatty liver disease, systematic review, health

## Abstract

**Objective:**

The oral microbiota is the second largest microbiota in the human body and has a significant impact on human health. Recent evidence suggests that dysbiosis of the oral microbiota may be associated with the development of metabolism-associated fatty liver disease (MAFLD). This review aimed to validate the relationship between oral microbial diversity and the development of MAFLD.

**Methods:**

A systematic evaluation was performed based on PRISMA guidelines. Three independent reviewers searched for relevant literature in several databases, including PubMed/Medline, Web of Science, and Scopus, with a search date ranging from the establishment of the databases to June 2024.

**Results:**

A total of 1278 publications were initially screened, including five cross-sectional studies, seven case-control studies, one cohort study, and one retrospective study. These studies included a total of 3335 patients with MAFLD, 254 patients with MASH, and 105 patients with liver cirrhosis. All 14 included studies concluded that there was a correlation or potential correlation between oral microbiota and MAFLD. Seven studies found that the composition of the oral microbiota in MAFLD patients differed from that of healthy controls, and specific oral bacteria may be associated with an increased incidence of MAFLD. At the *phylum* level, several studies found differences in the abundance of the *phyla Firmicutes*, *Proteobacteria*, and *Clostridia* compared to healthy controls. Additionally, a study on oral fungi found significant differences in the phyla *Proteobacteria* and in the genus *Staphylococcus* between patients with MAFLD and healthy controls. At the genus level, *Porphyromonas* was studied most frequently, with all 8 studies identifying infection with *Porphyromonas* as a significant risk factor for pathological progression in MAFLD. Furthermore, a dysbiosis in the ratio of *Porphyromonas gingivalis./Porphyromonas* anomalies may be an important marker of MAFLD progression.

**Conclusion:**

There is an important association between the diversity of oral microbiota composition and MAFLD. This finding suggests the importance of oral health assessment and monitoring for the prevention or intervention of MAFLD.

## 1 Introduction

Metabolic dysfunction-associated fatty liver disease (MAFLD) is a chronic liver disease linked to metabolic stress, resulting from overnutrition and insulin resistance in genetically susceptible individuals (Eslam et al., 2022). According to the diagnostic criteria set out in the 2020 International Consensus Statement of Experts, MAFLD is characterized by the presence of fat accumulation and metabolic dysfunction in the liver. It encompasses the full spectrum of the disease, from simple steatosis without inflammation and fibrosis to stage 4 fibrosis. MAFLD affects up to one-third of the world’s population (Eslam et al., 2022), and its prevalence is increasing in line with the rising rates of type 2 diabetes mellitus and obesity. As the disease progresses, MAFLD can advance to MASH (Metabolic Associated Steatohepatitis), characterized by hepatic inflammation and fibrosis, which significantly raises the risk of cirrhosis and hepatocellular carcinoma. MASH is a more severe form of MAFLD and is a crucial stage in the disease spectrum, often used as a marker for liver disease progression. If left unchecked, MASH can lead to end-stage liver disease, liver cancer, and death, and it may contribute to cardiometabolic disease.

MAFLD and MASH have been shown to be associated with obesity (Gutiérrez-Cuevas et al., 2021), insulin resistance (IR) (Sakurai et al., 2021), glucose abnormalities (Xue et al., 2022), and oxidative stress (Clare et al., 2022). Several recent studies have suggested that the human microbiota plays an important role in the development of MAFLD and its progression to MASH, not only through changes in the composition and relative abundance of gut microbiota but also through dysbiosis of the oral microbiota (Cani, 2018; Flemer et al., 2017). Dysregulation of the oral microbiota may further exacerbate hepatic inflammation, contributing to the transition from simple steatosis to MASH, emphasizing the need to study the role of oral bacteria in disease progression.

The oral microbiota is the second most diverse and numerous microbiota in the human body (Deo and Deshmukh, 2019; Suárez et al., 2021), containing approximately 700 species that form a complex ecological community. This microbiome includes a highly diverse array of viruses, fungi, bacteria, archaea, and protozoa (Caselli et al., 2020). As the oral cavity is considered the main entrance to the body, the microbiota within this ecological niche is likely to affect all parts of the body. When dysbiosis occurs in oral bacteria, it can lead to chronic inflammation, immune dysregulation, and damage to the oral mucosa, using it as a conduit to reach the bloodstream (Suárez et al., 2020). Dysbiosis of the oral microbiota has been associated with the development and progression of MAFLD diseases (Hajishengallis and Chavakis, 2021; Kuraji et al., 2021).

In recent years, there has been an increasing number of studies on the relationship between oral microbiota and MAFLD. One animal study suggested that endotoxemia caused by *P.gingivalis* is a significant risk factor for the development of MAFLD, and that altered glucose/lipid metabolism may contribute to the progression of the disease (Wang et al., 2022). Additionally, studies have shown that the oral microbiota remains dynamic and is influenced by different lifestyles, such as diet, stress, tobacco consumption, and systemic conditions, which alter the composition and characterization of the oral microbiota, thereby affecting disease progression in MAFLD (Baker and Edlund, 2019; Lee et al., 2021). Given that the oral cavity is a complex microbiological environment, there is a need to further understand the changes in the oral microbial community in the pathological state of MAFLD.

This is the first systematic review to assess the relationship between oral microbiota and MAFLD, The purpose of this article is to explore and compare the composition and diversity of oral microbiota in individuals with MAFLD, drawing insights from published studies to expand knowledge about the role of oral microorganisms in the progression of the disease.

## 2 Materials and methods

### 2.1 Protocol and registration

This is a systematic review that was registered with the International Prospective Register of Systematic Reviews and was reported based on the PRISMA statement (Chandler et al., 2019; Stewart et al., 2015). Details of the review protocol were registered on the PROSPERO International Prospective Register of Systematic Reviews in July 2024 (http://www.crd.york.ac.uk/prospero/, registration number: CRD42024531033).

### 2.2 Eligibility criteria

Literature search was performed according to the PICOS principles. POPULATION: Adult patients diagnosed with MAFLD. Intervention: detection of oral microbiota in the presence of MAFLD. Control: adult population without MAFLD. RESULTS: Association between oral microbiota composition and MAFLD. STUDY DESIGN: Studies evaluating the existence of an association between oral microbiota and the prevalence of MAFLD, the study design could be a case-control study, a cohort study or a cross-sectional study. Exclusion criteria included reviews, conference abstracts, case reports, etc., in which studies did not provide clear data for analyses of the association between oral microbiota and MAFLD.

### 2.3 Information sources and search strategy

Three independent researchers searched the literature from Pubmed//Medline, Web of Science, Scopus, and the Cochrane Library using the following keyword terms: oral microorganisms, MAFLD, oral microbiota, non-alcoholic fatty liver disease. The search was performed up to 10 June 2024.The search strategy was as follows. The search strategy is shown in **Table 1**.

**Table 1:** Search strategy in each electronic database.

Pubmed
**#1**:(((((“Oral Microorganisms”[MeSH]) OR (“Oral Microorganisms”)) OR (“Oral Microbiota”[MeSH])) OR (“Oral Microbiota”))) **#2**:(((((“MAFLD”[MeSH]) OR (“MAFLD”)) OR (“MAFLD”[MeSH])) OR (“MAFLD”)) OR (“MASH”[MeSH]) OR (“MASH”)) OR (“Non-alcoholic Fatty Liver Disease”[MeSH]) OR (“Non-alcoholic Fatty Liver Disease”)) **#3**:(((“Microbiome”[MeSH]) OR (“Microbiome”)) OR (“Liver Cirrhosis”[MeSH])) OR (“Liver Cirrhosis”) **#4**:1# AND 2# AND 3#
Web of Science
**#1**:TS=(“Oral Microorganisms” OR “Oral Microbiota”) **#2**:TS=(“MAFLD” OR “MAFLD” OR “MASH” OR “Non-alcoholic Fatty Liver Disease”) **#3**:TS=(“Microbiome” OR “Liver Cirrhosis”) **#4**:1# AND 2# AND 3#
Scope
**#1**:TITLE-ABS-KEY(“Oral Microorganisms” OR “Oral Microbiota”) **#2**:TITLE-ABS-KEY(“MAFLD” OR “MAFLD” OR “MASH” OR “Non-alcoholic Fatty Liver Disease”) **#3**:TITLE-ABS-KEY(“Microbiome” OR “Liver Cirrhosis”) **#4**:1# AND 2# AND 3#
The Cochrane Library
**#1**:Oral Microorganisms **#2**:MAFLD **#3**:Oral Microbiota **#4**:MAFLD **#5**:Microbiome **#6**:Non-alcoholic Fatty Liver Disease **#7**:MASH **#8**:Liver Cirrhosis **#9**:{or #1-#2} **#10**:{or #3-#6} **#11**:{or #7-#8} **#12**:{and #9 and #10 and #11}

### 2.4 Data collection process

The included literature was analyzed and data extracted by two independent researchers. The included literature was imported into NoteExpress software after literature search to remove duplicates, followed by the first round of screening by reading the title and abstract, and then reading the full text to complete the second round of screening. The extracted data included first author’s name, country, journal, year of publication, number of patients, patient’s age, oral diagnosis, type of microbiota analysis, sample extraction, testing method and main results. In case of disagreement during this period, a third researcher was involved in the decision.

### 2.5 Quality assessment of included studies

Depending on the type of design of the study, quality was evaluated using the Joanna Briggs Institute (JBI) Analytic Cross-Sectional Study Critical Appraisal Checklist (Moola et al., 2017).The JBI scale is a tool used to assess the quality of a cross-sectional study in terms of the sample frame, participant selection, undertakings and description of the results, confounding factor considerations, measurement instruments, data collection methods, statistical analysis methods, and interpretation of the results, respectively, for study quality were developed. According to the JBI tool, each study was categorized as: ‘High Quality’ (HQ) - high scores in all assessed domains; ‘Moderate Quality’ (MQ) - good performance in most domains with minor deficiencies; “low quality” (LQ)-poor performance in multiple domains and questionable credibility; and “very low quality” (VQ)-serious quality deficiencies; “Distal Quality” (UQ)-Some domains are scored unclearly or with questionable credibility. When using the JBI scale, the quality of the study was assessed on an item-by-item basis and scores were recorded (e.g., “yes,” “no,” or “unclear”) to synthesize the quality of the study. Two authors assessed each study independently and any disagreements were discussed with a third researcher.

## 3 Results

### 3.1 Literature search

**Figure 1:**
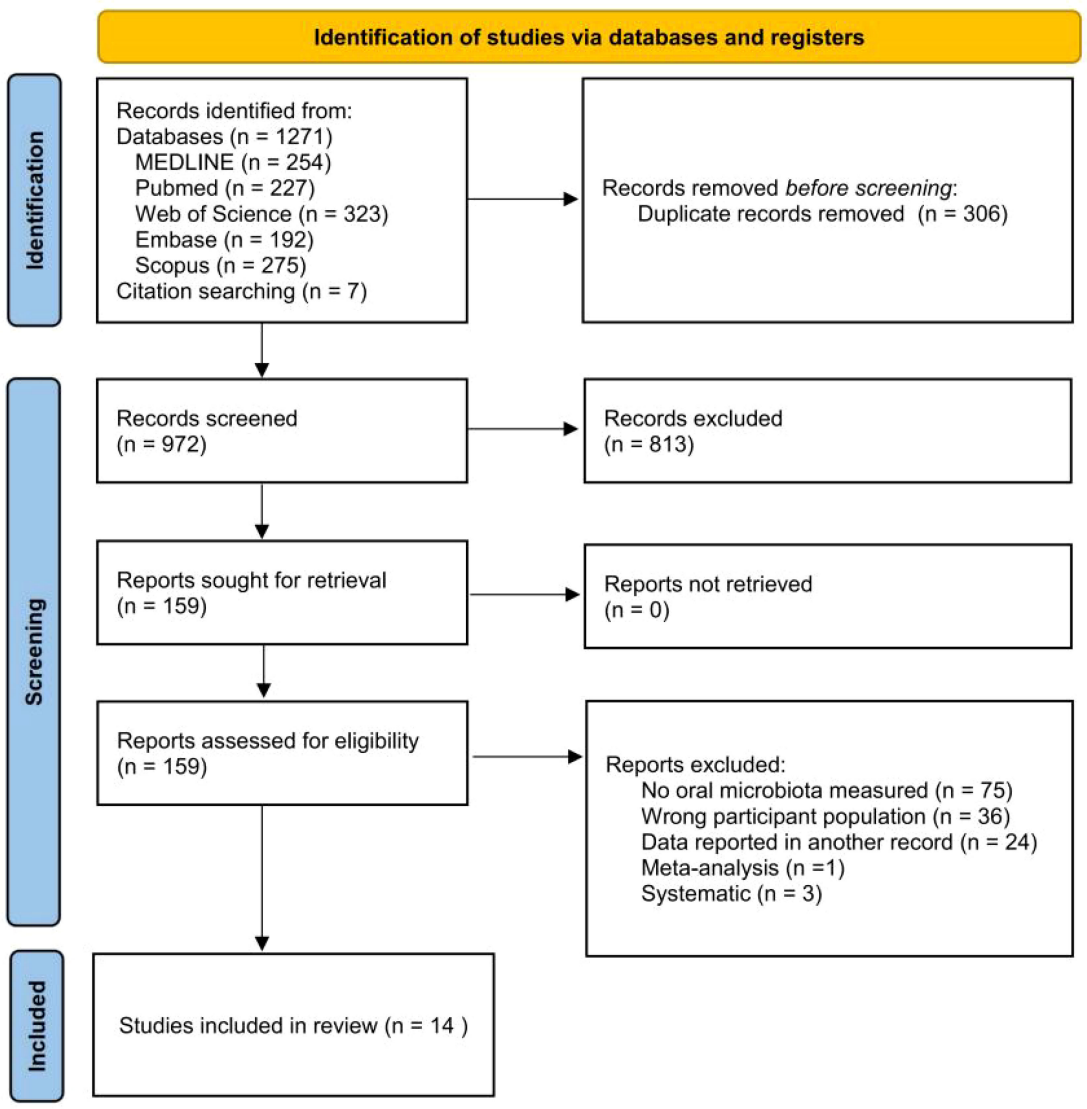
PRISMA flowchart diagram. From Page et al. (2021).

The search strategy is shown in **Figure 1**, and after the first round of search, a total of 1,271 studies were retrieved from Medline (n = 254), Pubmed (n = 227), Web of Science (n = 323), Embase (n = 192), and Scopus (n = 275), and 7 studies were retrieved by other methods. After deletion of duplicates, 965 studies remained. By reading the titles, abstracts, and according to the inclusion and exclusion criteria, 152 studies remained, and further reading of the full text resulted in the inclusion of 14 studies.

### 3.2 Description of the studies

The 14 included studies were published between 2012 and 2024 and consisted of 5 cross-sectional studies, 7 case-control studies, 1 cohort study, and 1 retrospective study. The studies were conducted in Japan (n=4), United Kingdom (n=2), China (n=4), Turkey (n=1), Iran (n=1), Denmark (n=1), and Germany (n=1). Regarding the type of liver disease, eight studies (57%) investigated the oral microbiological composition of patients with MAFLD (Alazawi et al., 2017; Zhao et al., 2020; Li et al., 2018; Nakahara et al., 2018; Niu et al., 2023; Sato et al., 2022; Wang et al., 2023; Zeybel et al., 2021), four studies investigated the status of oral microbiology in patients with MASH (Matsui et al., 2024; Nakahara et al., 2018; Pischke et al., 2023; Takuma et al., 2023) and two studies investigated the composition of oral microbiology in patients with liver cirrhosis (Ghapanchi et al., 2020; Jensen et al., 2018). All studies assessed the oral microbiological status of the chronic liver disease population and included a total of 3335 patients with MAFLD, 254 patients with MASH and 105 patients with liver cirrhosis (see **Table 2**).

**Table 2:** General characteristics of the selected studies investigating the association between oral microbiota and MAFLD.

	Reference	Country	Study Design	Study Sample Size	Type of chronic liver disease	Report on Association Microbiota/Periodontal Status	Statistical Analysis
1	Li et al. (2017)	China	case-control	N=24(MAFLD) N=22(Healthy Adults)	MAFLD	Yes	Yes
2	Takuma et al. (2023)	United Kingdom	Cross-Sectional Pilot Study	N=60(MASH)	MASH	Yes	Yes
3	Jensen et al. (2018)	Denmark	Cross-Sectional Pilot Study	N = 21(liver cirrhosis)	liver cirrhosis	Yes	Yes
4	Nakahara et al. (2018)	Japan	Cross-Sectional Pilot Study	N=200(MAFLD)	MAFLD	Yes	Yes
5	Yoneda et al. (2012)	Japan	case-control	N=102 (MASH) N=48(MAFLD) N=60(Healthy Adults)	MASH MAFLD	Yes	Yes
6	Matsui et al. (2024)	Japan	Cross-Sectional Pilot Study	N=41(MASH) N=19(MASH-HCC)	MASH MASH-HCC	Yes	Yes
7	Pischke et al. (2023)	Germany	prospective study	N=32(MASH)	MASH	Yes	Yes
8	Sato et al. (2022)	Japan	Cross-Sectional Pilot Study	N=32(MAFLD)	MAFLD	Yes	Yes
9	Alazawi et al. (2017)	United Kingdom	Cross-Sectional Pilot Study	N=2930(MAFLD)	MAFLD	Yes	Yes
10	Ghapanchi et al. (2020)	Iran	Case control	N=84(liver cirrhosis) N=84(Healthy Adults)	liver cirrhosis	Yes	Yes
11	Zhao et al. (2020)	china	Case control	N=24(MAFLD) N=22(Healthy Adults)	MAFLD	Yes	Yes
12	Niu et al. (2023)	china	Case control	N=21(MAFLD) N=20(Healthy Adults)	MAFLD	Yes	Yes
13	Wang et al. (2023)	china	Case control	N=10(MAFLD) N=10(Healthy Adults)	MAFLD	Yes	Yes
14	Zeybel et al. (2021)	Turkey	Case control	N=46(MAFLD) N=10(Healthy Adults)	MAFLD	Yes	Yes

### 3.3 Quality assessment and risk of bias of included studies

According to JBI Critical Appraisal Checklist for Analytical Cross-Sectional, four studies fulfilled all required questions (Jensen et al., 2018; Matsui et al., 2024; Sato et al., 2022; Takuma et al., 2023). Two studies satisfied seven questions (Alazawi et al., 2017; Nakahara et al., 2018). The most unanswered questions were related to strategies to deal with confounding factors as shown in **Table 3**.

**Table 3:** JBI critical appraisal checklist for analytical cross-sectional studies.

Reference	Questions							
	1	2	3	4	5	6	7	8
Takuma et al. (2023)	Yes	Yes	Yes	Yes	Yes	Yes	Yes	Yes
Jensen et al. (2018)	Yes	Yes	Yes	Yes	Yes	Yes	Yes	Yes
Nakahara et al. (2018)	Yes	Yes	Yes	Yes	Unclear	Unclear	Yes	Yes
Matsui et al. (2024)	Yes	Yes	Yes	Yes	Yes	Yes	Yes	Yes
Sato et al. (2022)	Yes	Yes	Yes	Yes	Yes	Yes	Yes	Yes
Alazawi et al. (2017)	Yes	Yes	Yes	Yes	Yes	No	Yes	Yes

1.Were the criteria for inclusion in the sample clearly defined?; 2.Were the study subjects and the setting described in detail?; 3.Was the exposure measured in a valid and reliable way?; 4.Were objective, standard criteria used for measurement of the condition?; 5.Were confounding factors identified?;6.Were strategies to deal with confounding factors stated?; 7.Were the outcomes measured in a valid and reliable way?; 8.Was appropriate statistical analysis used?

Three studies attended to most requirements according to the JBI checklist for cohort studies, suiting eleven questions (Ghapanchi et al., 2020; Li et al., 2018; Zeybel et al., 2021). Three studies satisfied eight questions (Zhao et al., 2020; Pischke et al., 2023; Yoneda et al., 2012) (see **Table 4**). Two studies satisfied nine questions (Niu et al., 2023; Wang et al., 2023) (see **Table 4**).

**Table 4:** JBI critical appraisal checklist for analytical cohort studies.

Reference	Questions										
	1	2	3	4	5	6	7	8	9	10	11
Li et al. (2017)	Yes	Yes	Yes	Yes	Yes	Yes	Yes	Yes	Yes	Yes	Yes
Yoneda et al. (2012)	Yes	Yes	Yes	Yes	Yes	No	Yes	Yes	No	No	Yes
Pischke et al. (2023)	Yes	Yes	Yes	Yes	Yes	No	Yes	Yes	Unclear	Unclear	Yes
Ghapanchi et al. (2020)	Yes	Yes	Yes	Yes	Yes	Yes	Yes	Yes	Yes	Yes	Yes
Zhao et al. (2020)	Yes	Yes	Yes	Yes	Unclear	Yes	Yes	Yes	Unclear	Unclear	Yes
Niu et al. (2023)	Yes	Yes	Yes	Yes	Yes	Yes	Yes	Yes	Unclear	Unclear	Yes
Wang et al. (2023)	Yes	Yes	Yes	No	No	Yes	Yes	Yes	Yes	Yes	Yes
Zeybel et al. (2021)	Yes	Yes	Yes	Yes	Yes	Yes	Yes	Yes	Yes	Yes	Yes

1.Were the two groups similar and recruited from the same population?; 2.Were the exposures measured similarly to assign people to both exposed and unexposed groups?; 3.Was the exposure measured in a valid and reliable way?; 4.Were confounding factors identified?; 5.Were strategies to deal with confounding factors stated?; 6.Were the groups/participants free of the outcome at the start of the study (or at the moment of exposure)?; 7.Were the outcomes measured in a valid and reliable?; 8.Was the follow up time reported and sufficient to be long enough for outcomes to occur? 9. Was follow up complete, and if not, were the reasons to loss to follow up described and explored? 10. Were strategies to address incomplete follow up utilized? 11. Was appropriate statistical analysis used?

### 3.4 Oral microbiota and MAFLD

Eight studies investigated the association between oral microbiota and MAFLD, consistently finding correlations between specific phyla or genera of oral microbiota and the disease. Adamo et al. (2023) discovered that, compared to healthy controls, MAFLD patients had a higher relative abundance of *Firmicutes* and a lower abundance of *Bacteroidetes* in their saliva. Additionally, the proportions of *Clostridium*, *Porphyromonas*, and *Veillonella* were higher, while the proportions of *Prevotella* were significantly lower in the oral microbiota of MAFLD patients.

Nakahara et al. (2018) found that periodontal pathogens and intestinal bacteria do not directly influence hepatocellular carcinoma (HCC). However, HCC has a direct impact on the salivary periodontal bacterial species, including *Porphyromonas gingivalis, Tannerella forsythia, Fusobacterium nucleatum*, and *Prevotella intermedia.*

P.g infection is an important risk factor for pathological progression in MAFLD. Increase in the monounsaturated/saturated fatty acid ratio may be an important change that facilitates progression of MAFLD.

Sato et al. (2022) aimed to demonstrate a close association between MAFLD, particularly liver stiffness, and the presence of *Porphyromonas gingivalis*. Yumiko Nagao et al. Alazawi et al. (2017) found that the frequency of type 4 *Porphyromonas gingivalis* infection increased with the progression of fibrosis stages. Alazawi et al. (2017) identified that antibodies to two specific bacteria, *Selenomonas noxia* (OR=1.13) and *Streptococcus oralis* (OR=1.14), are closely associated with steatosis (OR=1.14).

Zhao et al. (2020) revealed increased abundances of bacteria associated with oral infections and decreased abundances of potentially beneficial aerobic bacteria. Niu et al. (2023) findings illustrate the potential correlation between core mycobiome and the development of MAFLD and could propose potential therapeutic strategies.

Zeybel et al. (2021) compared MAFLD patients with varying disease severity to healthy individuals and found significant differences in oral microbiota composition. Patients with mild MAFLD had significantly lower abundances of specific species in *Firmicutes* (*Porphyromonas* spp. and *Fusobacterium rectum*), anaplastic bacilli (*Porphyromonas przewalskii*), and actinomycetes (Matharomycetes). Moderate MAFLD patients showed increased abundances in *Firmicutes* (Fusobacterium) and decreased in anaplastic bacilli (*Campylobacter* and *Haemophilus pusillus*). Severe MAFLD patients exhibited reduced abundances in Anaplasma (*Porphyromonas endodontiae* and *Prevotella*), *Aspergillus* (*Haemophilus pusillus*), and increased *Actinobacteria* (*Actinobacillus johnsonii*). Additionally, both severe and moderate steatosis patients had significantly reduced sputum Haemophilus abundances compared to the healthy population (see **Table 5**).

**Table 5:** Outcomes of the selected studies investigating the association between oral microbiota and MAFLD.

Reference	Sample	Measurement Method	Microbiota Associate with MAFLD	Main Finding(s)	Alpha diversity analysis
Li et al. (2017)	Saliva	16S rRNA V3-V4 sequencing	Phyla:*Firmicutes*,*Bacteroidetes* Genera:fusobacterium, *Porphyromonas* and *Veillonella*	In the NAFLD group, the abundance of *Firmicutes* increased, while the abundance of other bacterial species decreased. It was speculated that this imbalance in salivary bacteria is associated with nonalcoholic fatty liver disease.	Diversity: No significant difference between MAFLD and controls Abundance: Increased abundance of the *Firmicutes*.
Takuma et al. (2023)	Saliva	16S rRNA sequencing	Bacterial examinations. Genera:*P. gingivalis*、 F. nucleatum、T. forsythia、T. denticola、P. intermedia	The salivary *P.gingivalis* and F. nucleatum ratios and the serum antibody titers cross-reacting with them were higher in the NASH-HCC group than in the NASH group. Additionally, F. nucleatum ratio in the saliva and the salivary IgA flow rate showed a negative correlation. Oral *P. gingivalis* and *F. nucleatum* were possibly associated with NASH-HCC pathogenesis, and salivary IgA levels were correlated with F. nucleatum.	Diversity: The MASH-HCC group was significantly different from the MASH group. Abundance: The abundance of *Clostridium perfringens* was higher in MASH-HCC compared to MASH group.
Sato et al. (2022)	Saliva	16S rRNA sequencing	Genera:*P. gingivalis*	It demonstrated that the group with ≥0.01% *P.gingivalis* had higher liver stiffness as measured by MRE than the group with <0.01% *P.gingivalis* in saliva.	——
Alazawi et al. (2017)	Saliva	16S rRNA sequencing	Genera:Eubacteriumnodatum and *Actinomyces* naeslundii、*Streptococcus* intermedius, *Streptococcus oralis*, *Streptococcus* mutans, *Fusobacterium nucleatum*, Parvimonas micra, *Tannerella forsythia*, *Treponema denticola*, Aggregatibacter actinomyce-temcomitans mix, Eikenella corrodens, *Selenomonas noxia*, *Veillonella* parvula and *Campylobacter* rectus	we identify an association between steatosis and peripheral antibodies to a member of the *Firmicutes* phylum, *Selenomonas noxia* and *Streptococcus oralis*.MAFLD is associated with periodontitis and that the association is stronger with signifi ant liver fibrosis	——
Jensen et al. (2018)	supragingival plaque	16S rDNA V1-V3 sequencing	Phyla: *Firmicutes*,*Bacteroidetes* and *Actinobacteria* Genera:*Streptococcus*, *Lactobacillus*, *Actinomyces*, *Prevotella*, *Cutibacterium*、*P. gingivalis*	Between cirrhotic patients and healthy controls, e.g., *Porphyromonas*, *Tannerella hypogenes*, and *Dentate Dense Spirochetes*, generally showed lower abundance. Differences at the portal level were observed between the phylum *Thickettsia* (cirrhotic patients) and the phylum *Aspergillus* (healthy controls).	Diversity: The cirrhosis group was significantly different from the healthy control group. Abundance: The cirrhosis group had a higher abundance of *Firmicutes*phyla and a lower abundance of *Ascomycetes* and Mycobacterium phyla compared to the healthy control group.
Ghapanchi et al. (2020)	Saliva	API20E	Genera:*Klebsiella pneumonia, Enterobacter cloacae, Acinetobacter sp, Raoultella sp, Pseudomonas aeruginosa, Providencia sp, Serratia* sp.	the difference for microbial population in saliva samples between participants with end stage liver disease and the healthy group was compared showing a variety of pathogens in these cases including, *Klebsiella pneumonia*, *Enterobacter cloacae*, *Acinetobacter sp*, *Raoultella sp*, *Pseudomonas aeruginosa*, *Providencia sp*, *Serratia* sp.	——
Nakahara et al. (2018)	Serum	ELISA to detect serum IgG	Genera:*P. gingivalis*	Periodontal pathogens and intestinal bacteria affecting hepatocellular carcinoma are not present. However, HCC directly affects the salivary periodontal bacterial species *Porphyromonas gingivalis*, *Tannerella congenita*, *Clostridium nucleatum* and *Prevotella intermedia.P.g* infection is an important risk factor for pathological progression in MAFLD. Increase in the monounsaturated/saturated fatty acid ratio may be an important change that facilitates progression of MAFLD.	——
Matsui et al. (2024)	Saliva	GENE PREP STAR PI-1200A	Phyla:*Actinomycetota*、*Bacillota*、*Bacteroidota*、*Campylobacter*ota、*Cyanobacteria*、*Desulfobacterota*、*Fusobacteriota*、*Patescibacteria*、*Pseudomonadota*、*Spirochaetota*、*Synergistetes*	The abundance of *Clostridium nucleatum* in saliva was observed to be higher in the MASH-HCC group than in the MASH group, and HCC directly influenced the salivary periodontal bacterial species *Porphyromonas aeruginosa*, *Tannatella congenita*, *Clostridium nucleatum*, and *Prevotella intermedia*HCC directly affected the periodontal bacterial species *P.gingivalis*, *Tannerella forsythia*, *Fusobacterium nucleatum*, and *Prevotella intermedia* in the saliva	Diversity: MASH-HCC was significantly lower than MASH group. Abundance: The abundance of *Clostridium* perfringens in the MASH-HCC group was higher than that in the MASH group.
Yoneda et al. (2012)	Saliva	PCR(detection of *Porphyromonas gingivalis* and other *periodontal bacteria* )	Genera:*P. gingivalis*、*Treponema denticola*, *Prevotella intermedia*, *Tannerella forsythia*,Aggregatibactor actinomycetemcomitans, Campyrobacter rectus	The prevalence of *Porphyromonas gingivalis* infection was significantly higher in patients with MAFLD than in healthy subjects. This result suggests that *Porphyromonas gingivalis* infection may be involved in the pathogenesis of MAFLD. This result suggests that *Porphyromonas gingivalis* infection may be an independent risk factor for MAFLD.	——
Pischke et al. (2023)	supragingival microbiota	16S rRNA sequencing	Genera:*P. gingivalis*、*Actinomyces*	Association between *Porphyromonas gingivalis* and concomitant *Actinobacillus actinomycetemcomitans* and severity of liver injury in MASH patients.	——
Zhao et al. (2020)	supragingival microbiota	16S rRNA V3-V4 sequencing	Phyla:*Bacteroidetes、Proteobacteria、Actinobacteria、Firmicutes、Fusobacteria、Patescibacteria、Epsilonbacteraeota and Spirochaetes*	The abundance of bacteria associated with oral infections was increased and the abundance of potentially beneficial aerobic bacteria was decreased in patients with MAFLD; the diversity of the supragingival microbiota was higher in the MAFLD group than in the control group.	Diversity: significant difference Abundance: Bacterial abundance:Increased Aerobic bacterial abundance:Decreased
Niu et al. (2023)	Saliva	16S rRNA V3-V4 sequencing	fungal microbiome	The abundance of intestinal flora in MAFLD patients showed a similar trend, with significantly lower community diversity in MAFLD patients compared to healthy individuals.*Ascomycetes* constituted the main dominant phyla in the oral fungal biome of MAFLD patients and healthy controls.	Diversity: no significant difference Abundance: significant difference
Wang et al. (2023)	Saliva	16S rRNA V3-V4 sequencing	Genera:*Howardella*、*Treponema*、*Desulfobulbus*、*Bulleidia*、*Propionibacterium*、*Filifactor*、*Eggerthia*、*Fretibacterium*、*Shuttleworthia*、*Roseburia*、*Neisseria*、*Capnocytophaga*	MAFLD group had a higher abundance of genera *Howardella*, *Treponema*, *Desulfobulbus*, *Bulleidia*, *Propionibacterium*, *Filifactor*, *Eggerthia*, *Fretibacterium*, *Shuttleworthia*, and *Roseburia*.	Diversity: no significant difference Abundance: significant difference
Zeybel et al. (2021)	Saliva	16S rDNA sequencing	Phyla:*Firmicutes*、*Bacteroidetes*, *Proteobacteria*, *Actinobacteria*	We observed that the abundance of Haemophilus sputorum was significantly reduced in the oral microbiota of subjects with both severe and moderate steatosis versus no steatosis.	Diversity: no significant difference Abundance: significant difference

### 3.5 Oral microbiota and MASH and liver cirrhosis

Four studies examined the oral microbiota in patients with MASH (Matsui et al., 2024; Nakahara et al., 2018; Pischke et al., 2023; Takuma et al., 2023), and two studies investigated the oral microbiota composition in patients with liver cirrhosis (Ghapanchi et al., 2020; Jensen et al., 2018). Takuma et al. (2023) (2023) compared the oral microbiota between MASH and liver cancer groups, finding that the proportions of *Porphyromonas gingivalis* and *Fusobacterium nucleatum* were significantly higher in the liver cancer group. Matsui et al. (2024) recruited 41 MASH patients and 19 MASH-HCC patients to analyze the periodontal bacteria and oral microbiota composition, discovering that liver cancer directly affected the levels of *P. gingivalis*, *Tannerella forsythia*, F. nucleatum, and *Prevotella intermedia* in saliva, although periodontal pathogens and oral microbiota did not directly impact liver cancer progression (Matsui et al., 2024). Jensen et al. (2018) found that patients with liver cirrhosis had generally lower abundances of *P. gingivalis*, T. forsythia, and *Treponema denticola* compared to healthy controls. Ghapanchi et al. (2020) reported significantly higher levels of *Escherichia coli* in patients with liver cirrhosis compared to healthy individuals (see **Table 5**).

## 4 Discussion

Oral microbiota, recognized as the second largest microbiota in the human body, significantly influences human health. Maintaining the homeostasis of oral microorganisms helps the body resist adverse external stimuli. In contrast, dysregulation can lead to both oral and systemic diseases (Qi et al., 2021). Studies have established a close association between oral microbes and the development of various conditions, including digestive diseases, cardiovascular diseases, bone-related diseases, and Alzheimer’s disease (Bastos et al., 2011; Hu et al., 2017). In recent years, the link between salivary oral microbiota and metabolic-associated fatty liver disease (MAFLD) has garnered increasing research attention. Most studies focus on elucidating the mechanisms of MAFLD, enhancing predictive diagnostics, and identifying potential interventional biomarkers through oral microbes (Åberg and Helenius-Hietala, 2022; Nagao et al., 2014). Given the critical role of oral microbiota in relation to MAFLD, this study provides a systematic review of the current literature, aiming to deepen the understanding of the possible relationship between oral health and MAFLD.

This review analyzed a total of 14 studies, which collectively involved 3,335 patients diagnosed with MAFLD, along with 254 patients with MASH and 105 patients with liver cirrhosis. Each study indicated a correlation or potential correlation between specific phyla or genera of oral microbiota and MAFLD. Notably, seven studies demonstrated that the composition of the oral microbiota in patients with MAFLD was distinct from that of healthy controls, suggesting a link between particular oral bacteria and an elevated risk of developing MAFLD. Regarding phyla, several studies reported significant differences in the abundance of *Firmicutes*, *Ascomycota*, and *Clostridia* in comparison to healthy controls (R. Li et al., 2017; Matsui et al., 2024; Nakahara et al., 2018; Takuma et al., 2023). Additionally, research focused on oral fungi found notable differences in the phyla *Ascomycota* and the genus *Staphylococcus* among individuals with MAFLD compared to healthy individuals. Among the genera studied, *Porphyromonas* emerged as a significant focus, with all ten studies indicating a relationship between *Porphyromonas* and the progression of MAFLD, highlighting its relevance in this area of investigation.

Oral microbial diversity is crucial for maintaining health and varies with the physiological state of the host (Giordano-Kelhoffer et al., 2022). Increased microbiome richness and diversity are recognized as markers of healthy ecosystems, particularly within oral microbial ecosystems (Falony et al., 2019). Alpha diversity analyses of oral microbiota, which include both richness and diversity dimensions, utilize commonly used richness indices such as Chao1 and ACE, and diversity indices such as Shannon and Simpson (L. Wang et al., 2019).Among the studies included in this review, eight conducted Alpha diversity analysis (Zhao et al., 2020; Jensen et al., 2018; Li et al., 2017; Matsui et al., 2024; Niu et al., 2023; Takuma et al., 2023; Wang et al., 2023; Zeybel et al., 2021), but results varied across studies. Five of these studies compared the oral microbiota of patients with MAFLD to that of healthy controls, with only one study showing a significant difference in diversity between the two groups. However, in terms of abundance, all five studies demonstrated that the diversity of the gingival microbiota was higher in the MAFLD group compared to the healthy control group. This discrepancy may be due to the sampling methodologies; four studies derived their samples from saliva, whereas the study showing a significant difference used supragingival microbiota. The oral cavity contains different sites that provide specific niches for microbial colonization characterized by varying oxygen levels, nutrient availability, and mechanical stress conditions. Consequently, the type of sample used could significantly impact the results when assessing the link between oral microbiota and MAFLD. Additionally, Alpha diversity analysis was performed on the MASH-HCC group and the MASH group, revealing significant differences between the two groups in terms of both diversity and abundance. The abundance of *C. nucleatum* was notably higher in the MASH-HCC group than in the MASH group, suggesting a potential role of *C. nucleatum* in the progression of NASH to hepatocellular carcinoma. In conclusion, while healthy subjects did not show differences in the diversity of salivary flora, they did show differences in the abundance of certain flora, such as Phylum *Firmicutes* and *Clostridium nucleatum*, compared to MAFLD patients. MASH shows differences in both diversity and abundance when compared to MASH-HCC. Given that sampling in the included studies was predominantly from saliva, with only two studies sampling from supragingival microbiota, further research is needed to validate the impact of different sampling methods on the differences observed in the flora.

Another finding of this review is that a dysregulated ratio of *Firmicutes* to *Bacteroidetes* may be an important marker of MAFLD progression (Stojanov et al., 2020). A lower ratio of these phyla is considered characteristic of oral and intestinal health. Previously, numerous studies have identified dysbiosis in the intestinal flora of patients with MAFLD and have suggested that this imbalance is associated with steatosis and fibrosis in the liver (Abenavoli et al., 2023; Jasirwan et al., 2021). Similarly, the dysregulation of the ratio of Bacteroides *Firmicutes*to *Bacteroides anthropophilus* was also confirmed in oral microbiology. Li et al. (2017) found this dysregulation in the oral microbiological analyses of patients with MAFLD and suggested that it was a contributing factor in the pathogenesis of MAFLD induced by the intestinal flora. Additionally, Zhao et al. (2020) observed a lower percentage of the *Firmicutes*phylum to anabolic phylum in the supragingival plaque of the control group compared to the MAFLD group (61.41% and 72.38%, respectively). Moreover, specific strains within the *Firmicutes*phylum, such as *Lactobacillus* and *Streptococcus*, were found to have increased abundance in patients with MAFLD, potentially exacerbating the metabolic imbalance and inflammatory response. Overall, these studies suggest that the dysregulation of the *Firmicutes* phylum to Anthrobacteria phylum ratio is not only a crucial hallmark of MAFLD but may also play a key role in its pathogenesis and progression by influencing the gut-hepatic axis mechanisms. Future studies should continue to explore the specific mechanisms of this disproportion and evaluate potential interventions to treat MAFLD by modulating both the gut and oral microbiota.

*Porphyromonas*, a Gram-negative anaerobic bacterium, is widely recognized as a primary causative agent of chronic periodontitis (Darveau, 2010). Beyond oral health, *Porphyromonas* is considered a confounding risk factor for systemic diseases such as cardiovascular disease, diabetes mellitus (DM), preterm birth, and rheumatoid arthritis (Figuero et al., 2011; Pizzo et al., 2010; Seymour et al., 2007; Wada and Kamisaki, 2010). Yoneda et al. (2012) discovered that the prevalence of *Porphyromonas* infections was significantly higher in patients with MAFLD than in healthy subjects. Similarly, Takuma et al. (2023) found that the proportion of *Porphyromonas* and *Clostridium nucleatum* in saliva was higher in the NASH-HCC group compared to the NASH group. Satsuki Sato observed that the group with ≥0.01% *Porphyromonas* in their saliva had a higher proportion of *Porphyromonas gingivalis* than the group with less than 0.01%. Additionally, the *Aeromonas gingivalis* group with less than 0.01% exhibited greater liver stiffness as measured by Magnetic Resonance Elastography (MRE).Several studies have demonstrated that oral administration of *Porphyromonas gingivalis* can lead to dysbiosis of the intestinal flora, decreased expression of ileocecal connexin genes, and impaired intestinal barrier function. This disruption may subsequently result in systemic endotoxemia, insulinemia, and hepatic steatosis (Ohtsu et al., 2019; Sohn et al., 2022). Furthermore, an increase in *Porphyromonas* DNA has been detected in the liver following oral administration of the bacteria. Collectively, this body of evidence suggests that *Porphyromonas gingivalis* infection may be an independent risk factor for MAFLD/MASH.

The potential translocation ability of the oral microbiota through the circulation system or following the flow of food and fluids into the digestive system could explain its presence in the gut and its role in MAFLD (Sun et al., 2020). Tonetti et al. (2013) mentioned that oral microbiota may contribute to the development of MAFLD through two mechanisms. First, oral microbiota might reach the intestines, disrupt the intestinal barrier, and affect the liver through the ‘gut-liver axis,’ leading to a chronic inflammatory response and promoting lipid deposition in the liver. Second, oral microbiota and their metabolites could enter the bloodstream via periodontal pockets, or the inflammatory reactions caused by oral bacteria might release inflammatory mediators into the bloodstream, contributing to systemic inflammation and liver damage.

The limitations of this systematic review include the relatively low number of participants and the small number of published studies meeting the inclusion criteria. While the studies employed various methods and sample types to analyze the oral microbiota, this variability should not necessarily be viewed as a limitation. Different regions of the oral cavity, such as saliva or supragingival plaque, can provide valuable insights, and the use of diverse detection methods—such as 16S rRNA sequencing or PCR—offers complementary advantages. For example, 16S rRNA sequencing, which relies on PCR amplification of specific ribosomal RNA fragments, is useful for broad bacterial community profiling, whereas other PCR methods may target specific microbial species. Each method has its own strengths, and a combination of approaches may be necessary in certain cases. Future research should focus on increasing sample sizes and exploring the complementary use of multiple methods to gain a more comprehensive understanding of the relationship between oral microbiota and MAFLD.

Many studies did not adequately address potential confounding factors that could influence the relationship between oral microbiota and MAFLD. These confounding factors include, but are not limited to, participants’ age, gender, dietary habits, smoking status, and overall health conditions. Failure to effectively control these confounding factors can lead to biased or spurious associations, affecting the reliability of the study results. For instance, some studies may not consider participants’ dietary habits, which can significantly impact the composition of the oral microbiota. To enhance the accuracy and reliability of future research, it is crucial to carefully identify and control these confounding factors and use appropriate statistical methods to adjust for these variables.

The type of sample used (e.g., saliva, supragingival plaque) can significantly influence the study results, as different oral sites may harbor distinct microbial communities. The oral cavity is a complex environment where various sites have different oxygen levels, nutrient availability, and mechanical stress conditions, leading to variations in microbial distribution and species. Therefore, differences in sampling techniques add complexity to interpreting the results. For example, saliva samples may better reflect the overall oral microbiota, whereas supragingival plaque samples may better represent local microbial communities. To obtain more reliable results when assessing the link between oral microbiota and MAFLD, future research should standardize sampling techniques and consider the characteristics of microbial communities at different oral sites.

To our knowledge, this is the first literature systematic review that evaluated the possible relationships between oral microbiota and MAFLD. They confirmed our hypothesis that there is relationship between the composition and diversity of oral microbiota with MAFLD. Further experimental investigations are necessary to confirm the conclusions presented in this systematic review. These results point to the importance to develop studies that could considerably aid the clinical management of MAFLD. Therefore, Assessment of the oral microbiota’s helps predict and prevent MAFLD.

## 5 Conclusions

The link between the diversity of oral microbiota and MAFLD underscores the critical role of assessing and monitoring oral health in the prevention and management of MAFLD. This connection highlights the potential benefits of prioritizing oral healthcare in strategies aimed at addressing MAFLD.
